# *Sorbus alnifolia* protects dopaminergic neurodegeneration in *Caenorhabditis elegans*

**DOI:** 10.1080/13880209.2016.1251468

**Published:** 2016-12-09

**Authors:** Se-Myeong Cheon, Insoo Jang, Myon-Hee Lee, Dae Keun Kim, Hoon Jeon, Dong Seok Cha

**Affiliations:** a College of Pharmacy, Woosuk University, Jeonbuk, Republic of Korea;; b Department of Korean Internal Medicine, Woosuk University, Jeonbuk, Republic of Korea;; c Department of Medicine, Brody School of Medicine at East, Carolina University, Greenville, NC, USA

**Keywords:** Parkinson’s disease, neurotoxicity, tyrosine hydroxylase, food sensing, lifespan-extension

## Abstract

**Context:** The twigs of *Sorbus alnifolia* (Sieb. et Zucc.) K. Koch (Rosaceae) have been used to treat neurological disorders as a traditional medicine in Korea. However, there are limited data describing the efficacy of *S. alnifolia* in Parkinson’s disease (PD).

**Objective:** This study was conducted to identify the protective effects of the methanol extracts of *S. alnifolia* (MESA) on the dopaminergic (DA) neurodegeneration in *Caenorhabditis elegans*.

**Materials and methods:** To test the neuroprotective action of MESA, viability assay was performed after 48 h exposure to 1-methyl-4-phenylpyridine (MMP^+^) in PC12 cells and *C. elegans* (400 μM and 2 mM of MMP^+^, respectively). Fluorescence intensity was quantified using transgenic mutants such as BZ555 (Pdat-1::GFP) and and UA57 (Pdat-1::GFP and Pdat-1::CAT-2) to determine MESA’s effects on DA neurodegeneration in *C. elegans*. Aggregation of α-synuclein was observed using NL5901 strain (unc-54p::α-synuclein::YFP). MESA’s protective effects on the DA neuronal functions were examined by food-sensing assay. Lifespan assay was conducted to test the effects of MESA on the longevity.

**Results:** MESA restored MPP^+^-induced loss of viability in both PC12 cells and *C. elegans* (85.8% and 54.9%, respectively). In *C. elegans*, MESA provided protection against chemically and genetically-induced DA neurodegeneration, respectively. Moreover, food-sensing functions were increased 58.4% by MESA in the DA neuron degraded worms. MESA also prolonged the average lifespan by 25.6%. However, MESA failed to alter α-synuclein aggregation.

**Discussion and conclusions:** These results revealed that MESA protects DA neurodegeneration and recovers diminished DA neuronal functions, thereby can be a valuable candidate for the treatment of PD.

## Introduction

Parkinson’s disease (PD) is a major progressive neurodegenerative disorder and the prevalence rate of PD for those aged 65 and above is more than 1% (Dawson & Dawson 2002). The main symptoms of PD are tremor, bradykinesia and rigidity resulting from the death of dopaminergic (DA) neurons in the substantia nigra of the midbrain as well as Lewy body accumulation. The Parkinson’s symptoms can be managed with several medications which regulate dopamine level in the brain or mimic the dopamine effect. However, to date, there is no available treatment that cures or slows down the progress of this disease.

Although the pathogenesis of PD is not completely clear, accumulating evidence suggests that mitochondrial dysfunction results in increased reactive oxygen species (ROS) production and decreased ATP synthesis possibly generate the loss of dopaminergic cells (Trimmer & Bennett 2009). Previous studies revealed that 1-methyl-4-phenylpyridinium ion (MPP^+^) which inhibits mitochondrial complex I activity can induce PD-like symptoms in humans and animal models (Langston et al. 1983; Schmidt & Ferger 2001). In addition, high levels of mitochondrial DNA deletions and mutations were observed from PD patients (Parker & Parks 2005; Bender et al. 2006). Therefore, preventing mitochondrial dysfunction or reducing oxidative stress has been considered as an attractive therapeutic target for PD treatment. Recently, new drugs originating from natural products have received much attention, and many plant-derived compounds show neuroprotective properties in animal PD models. Thus, exploration of ethnopharmacological treatments may be a cost-effective and promising way for developing novel drugs for PD.

Recently, despite their lack of evolutionary complexity, *Caenorhabditis elegans* has become a popular experimental model system for screening drugs relevant to neurodegenerative diseases, including PD with several advantages (Schmidt et al. 2007). This simple organism has eight DA neurons that can be easily visualized by expressing a fluorescent protein, and thus, allows identification of morphological changes during neurodegenerative processes. Moreover, the components and functions of mammalian DA system are highly conserved in this simple organism.

*Sorbus alnifolia* (Sieb. et Zucc.) K. Koch (Rosaceae) is also known as Alder-leafed White beam or Korean White beam. The bark of *Sorbus* species are believed to possess strong therapeutic potential regarding neurological disorders, such as stroke and neurological pain. However, there is no scientific evidence to support this traditional usage. Hence, this study was undertaken to verify the protective role of the twigs of *S. alnifolia* on the pathogenesis of PD. In the present study, we tested neuroprotective potential of *S. alnifolia* against MPP^+^-induced toxicity in both PC12 cells and wild-type *C. elegans*. We also investigated the effects of *S. alnifolia* on the DA neurodegeneration and DA-related functional characteristics in *C. elegans*.

## Materials and methods

### Plant material and extraction

The plant materials were purchased from Kangwonyakcho (Kangwon, South Korea) in March 2012. The plant was identified by Dr. Dae Keun Kim (College of Pharmacy, Woosuk University). A voucher specimen (WH086) has been deposited at the Department of Oriental Pharmacy, College of Pharmacy, Woosuk University. The dried stems and twigs of the plant (600 g) were extracted using 6000 mL of MeOH with 2 h sonication. The resultant methanol extract (MESA) was concentrated into 28.1 g (yield 4.68%) using a rotary evaporator. Then, MESA was lyophilized and stored at −20 °C for further use. The MESA was initially dissolved in D.W. and diluted with M9 buffer to the appropriate concentration.

### Cell culture condition and cell viability assay

NGF-differentiated rat pheochromocytoma cells (PC12 cells) were kindly provided by Dr. Eun Joo Bae (College of Pharmacy, Woosuk University) and maintained in Dulbecco’s modified Eagle’s medium (DMEM) supplemented with 10% heat-inactivated fetal bovine serum, 100 U/mL penicillin and 100 μg/mL streptomycin in a 5% CO_2_ incubator at 37 °C. PC12 cells were plated at a density of 1.5 × 10^5^ cells/well in 96-well plates and incubated for 24 h. Then, the cells were pretreated with MESA and incubated for 2 h. After the pretreatment period, 400 μM MPP^+ ^was added to the culture medium. After an additional 48 h incubation, MTT assay was performed to determine the cell viability.

### *C. elegans* culture and maintenance

Worms were grown on the nematode growth medium (NGM) agar plate and maintained at 20 °C as described previously (Brenner 1974). Living *Escherichia coli* bacteria (OP50) was added to the surface of NGM plates for providing the food source. In this study, wild-type Bristol N2 and transgenic strains including BZ555 (egIs1, Pdat-1::GFP), UA57 (baIs4, Pdat-1::GFP; Pdat-1::CAT-2), CB1112 (e1112, cat-2 loss of function), NL5901 (pkIs2386, unc-54p::alphasynuclein::YFP) were used. Bristol N2 (wild-type) and *E. coli* OP50 strain were kindly provided by Dr. Myon-Hee Lee (East Carolina University, NC, USA). Transgenic strains were obtained from the Caenorhabditis Genetic Center (CGC; University of Minnesota, Minneapolis, MN).

### Treatment of worms with MPP^+ ^and viability assay

Age-synchronized L1 larvae were transferred to 96-well plate at an average of 15 worms/well in a 40 μL of liquid culture solution with concentrated OP50. Then, MPP^+ ^(2 mM) and various concentrations of MESA were added to make a final volume of 50 μL per well. After 48 h incubation, the worm viability was examined under the stereo-microscope. Test worms were considered dead when they failed to respond to prodding with the tip of a platinum wire. Each test was performed at least three times.

### Analysis of dopaminergic neurodegeneration

Age-synchronized L1 larvae of BZ555 (Pdat-1::GFP) worms were incubated with MPP^+ ^(1 mM) alone or in the presence of various concentrations of MESA in the 96-well plate. L1 larvae of UA57 (Pdat-1::GFP and Pdat-1::CAT-2) worms were transferred to 96-well plate and incubated in the presence or absence of MESA. On the 5th day of adulthood, either the MPP^+^-treated BZ555 worms or the UA57 worms were immobilized with 200 mM sodium azide and mounted onto 2% agarose pads. The DA neurodegeneration was microscopically observed using a fluorescence microscope (Nikon Eclipse Ni-u, Japan). DA neurons were counted in each survived animal by inspecting the GFP fluorescence. The data were obtained from four independent experiments and expressed as the percentage of normal controls. The fluorescence signals of DA neuron were quantified with Image J software (NIH, Bethesda, MD) and also photographed at 200 × magnification.

### Analysis of α-synuclein aggregation

Effects of MESA on the α-synuclein aggregation was evaluated using NL5901 strain (pkIs2386, unc-54p::alphasynuclein::YFP) as reported previously (Jadiya et al. 2011). Briefly, age-synchronized worms were washed three times with M9 buffer to get rid of remaining bacteria and mounted onto 2% agarose pads. Then, worms were immobilized with 200 mM sodium azide. To monitor the α-synuclein aggregation, YFP protein was microscopically visualized and photographed using a fluorescence microscope (Nikon Eclipse Ni-u, Japan). The fluorescence signals were quantified in each worm with Image J software (NIH, Bethesda, MD).

### Food sensing assay

Well-fed worms with intact DA neural circuitry moved slower in the presence of bacterial food than in its absence. This basal slowing response (or food-sensing response) was assayed as described by Sawin et al. (2000). Briefly, food-containing plates were prepared by spreading the bacterial food OP50 and together with non-coated plates, they were incubated at 37 °C overnight and cooled to room temperature prior to the assay. Age-synchronized worms were transferred from their food-containing culture plate to a non-food plate and washed in M9 buffer (100 mM NaCl, 10 μg/mL cholesterol, 50 mM potassium phosphate, pH 6.0) three times to get rid of the remaining food. For the food sensing assay, travel distances in both food-coated plate and non-coated plated were measured and basal slowing responses were calculated.

### Lifespan assay

The lifespan assays were performed using N2 and NL5901 strains at 20 °C. To obtain age-synchronized nematodes, eggs were transferred to NGM plate in the absence or presence of 250 μg/mL of MESA after embryo isolation. The number of worm was daily counted under the stereo-microscope. Test worms were considered dead when they failed to respond to prodding with the tip of a platinum wire. The worms were transferred to fresh NGM plate every 2 days.

### Data analysis

The data from the lifespan assay were plotted using the Kaplan–Meier analysis and statistical significance was analyzed by the log-rank test. Other data were presented as mean ± standard deviation or standard error of the mean, as indicated. Statistical significance of differences between the the control and treated groups were analyzed by one-way analysis of variance (ANOVA).

## Results

### MESA reduces MPP^+^-induced neurotoxicity in vitro and in vivo

**Figure 1.**
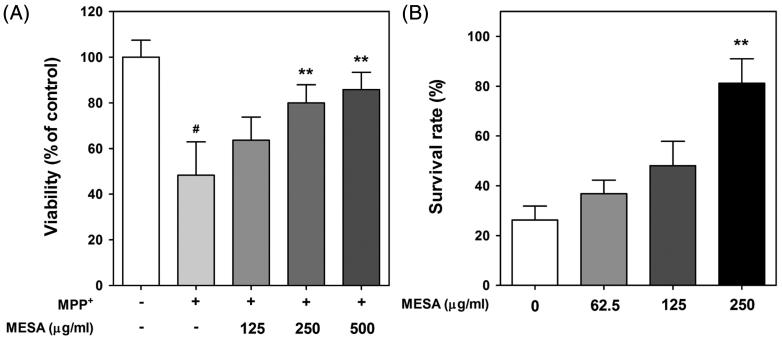
Protective effects of MESA against MPP^+^-induced toxicity. (A) PC12 cells plated at a density of 1.5 × 10^5^ cells/well and pretreated with MESA. After 2 h incubation, 400 μM of MPP^+ ^was added to the culture medium and incubated for 48 h. MTT assay was performed to determine the cell viability. (B) Worms were treated with 2 mM of MPP^+ ^for 48 h and MESA was added 30 min before MPP^+ ^treatment. Data are expressed as the mean ± S.D. and results are obtained from three independent assays with triplicate determinations. Significance of difference between MESA treatment and MPP^+^-treated control was determined by a one-way ANOVA, followed by a Tukey mean comparison *post hoc* test. #*p* < 0.05 compared with vehicle alone. **p < 0.01 compared with MPP^+^-treated control.

In case of *in vitro* study, MPP^+^-mediated oxidative damage in PC12 cells has been widely used as a cellular model to explore the possible anti-Parkinson agent (Kook et al. 2011; Li et al. 2013). The cell viability of PC12 cells was determined by MTT assay to verify whether MESA can reduce MPP^+^-induced damage. The relative values (% of control in mean ± S.D.) of MPP^+^-treated cells were 48.27 ± 16.95%. In this study, MESA treatment effectively protected MPP^+^-induced cytotoxicity in a dose-dependent manner in NGF-differentiated PC12 cells. The values of viability of MESA-treated cells were 63.61 ± 11.76, 79.93 ± 9.22 and 85.83 ± 8.74% at 125, 250 and 500 μg/mL, respectively (Figure 1(a)). However, MESA alone did not change the cell viability, indicating the lack of toxicity at the treatment concentration (Data not shown). To test whether MESA has protective potential against MPP^+^-induced toxicity *in vivo*, survival assay was carried out using *C. elegans* model system. After MPP^+ ^exposure, substantial worm death occurred. The relative value of survival rate was 26.25 ± 6.87 after the treatment of 2 mM of MPP^+^. As shown in Figure 1(b), pretreatment of MESA at the dosage of 62.5, 125, and 250 μg/mL significantly restored MPP^+^-induced loss of viability by 10.62%, 21.85%, and 54.93%, respectively. However, MESA alone did not change the survival rate of worms. These observed results indicated that MESA has protective potential against MPP^+^-mediated neurotoxicity.

### MESA protects DA neurodegeneration in *C. elegans*

**Figure 2.**
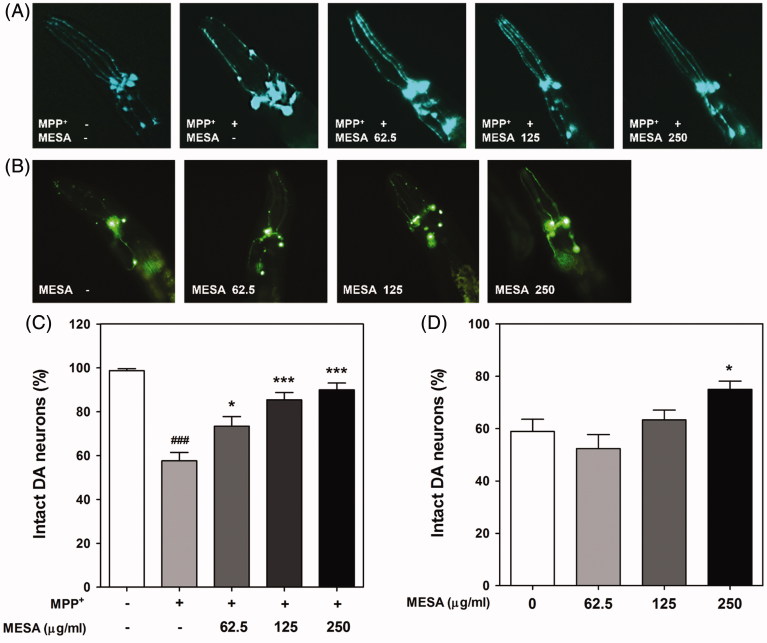
Protective effects of MESA on the DA neurodegeneration in *C. elegans*. GFP expression patterns of MPP^+^-treated transgenic strain BZ555 (Pdat-1::GFP) (A) and UA57 (Pdat-1::GFP and Pdat-1::CAT-2) (B). The fluorescence signals of DA neuron were photographed at 200 × magnification using fluorescence microscope. All eight DA neurons were counted in each survived animal by inspecting the GFP fluorescence of BZ555 (C) and UA57 (D), respectively. Data are expressed as the mean ± S.E.M. and results are obtained from three independent assays. Significance of difference between MESA treatment and control was determined by a one-way ANOVA. ###*p* < 0.001 compared with vehicle alone, *p < 0.05 and ***p < 0.001 compared with control.

To investigate whether MESA could reduce MPP^+^-induced DA neurodegeneration, normality of DA neurons was analyzed using transgenic strain BZ555, expressing GFP in the DA neurons. In this study, control worms exhibited bright GFP fluorescence in the cell bodies and dendrites from the nerve ring to the tip of nose. In contrast, as can be seen in Figure 2(a), 4 days of MPP^+ ^(1 mM) exposure to worms caused a typical MPP^+^-induced degeneration of DA neurons. The MESA significantly elevated the resistance of DA neurons to MPP^+ ^at all treated concentration of MESA (Figure 2(c)). Since over-expression of *cat-2* gene, a tyrosine hydroxylase homolog, is believed to induce DA neurodegeneration, we further checked MESA’s possible protective effects on the loss of DA neurons in the CAT-2 protein over-expressing transgenic strain UA57. Excessive CAT-2 protein in DA neurons causes degeneration at all developmental stages, but becomes more pronounced as animals age. On the 7th day of adulthood, DA neurons of UA57 worms were degraded significantly (Figure 2(b)). The MESA was found to suppress this DA neurodegeneration ∼16.07% at 250 μg/mL (Figure 2(d)). These results showed that MESA has potent protective properties on the DA neurodegeneration induced both chemically and genetically.

### MESA failed to prevent α-synuclein aggregation in *C. elegans*

**Figure 3.**
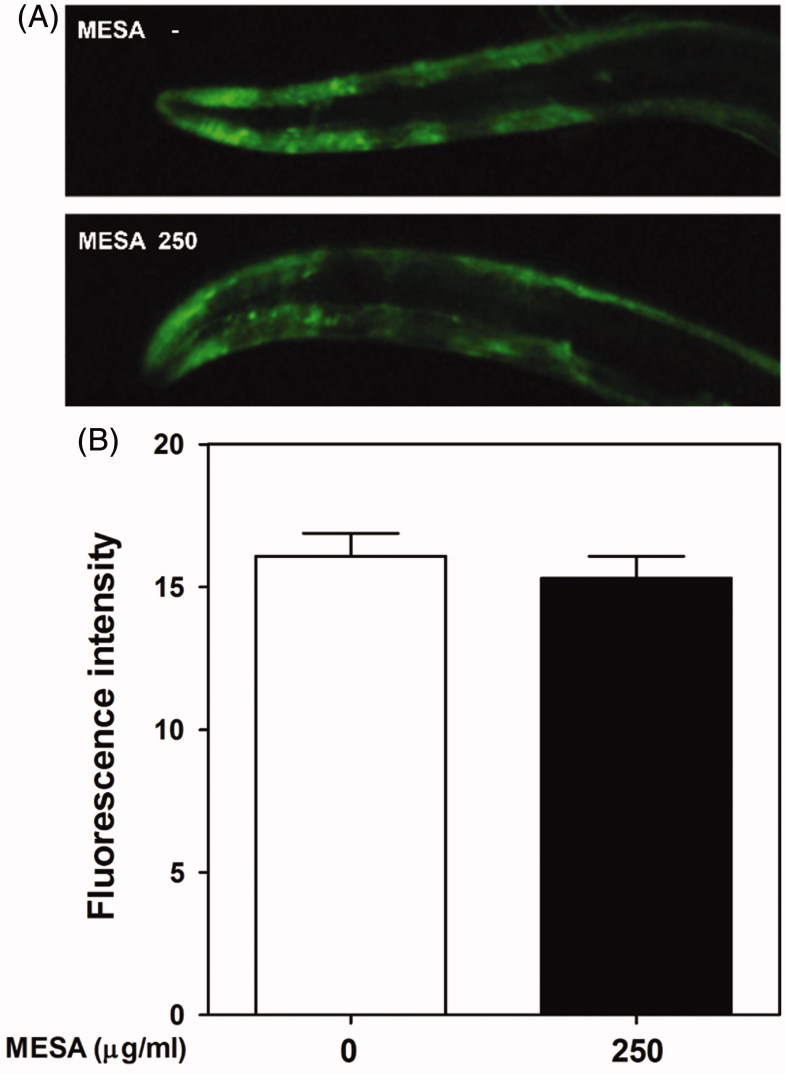
Effects of MESA on the α-synuclein aggregation in *C. elegans*. Aggregation of α-synuclein protein was observed using transgenic strain NL5901 (pkIs2386, unc-54p::alphasynuclein::YFP). (A) YFP protein was microscopically visualized and photographed using a fluorescence microscope. (B) The fluorescence intensity was quantified with Image J software. Data are expressed as the mean ± S.E.M. and results are obtained from three independent assays.

Previous discoveries have noted that pathological α-synuclein aggregation is associated with DA neurodegeneration (Farrer et al. 2004). Since *C. elegans* does not express a homolog of α-synuclein, we used transgenic strain NL5901 expressing human α-synuclein to investigate possible involvement of α-synuclein attenuation in the MESA-mediated DA neuroprotection. In this work, we could not detect significant differences in YFP fluorescence signals between MESA-treated and vehicle-treated worms, suggesting no effect of MESA on the α-synuclein accumulation (Figure 3(a,b)).

### MESA restores DA-specific behaviuoral dysfunction in the MPP^+^-treated *C. elegans*

**Figure 4.**
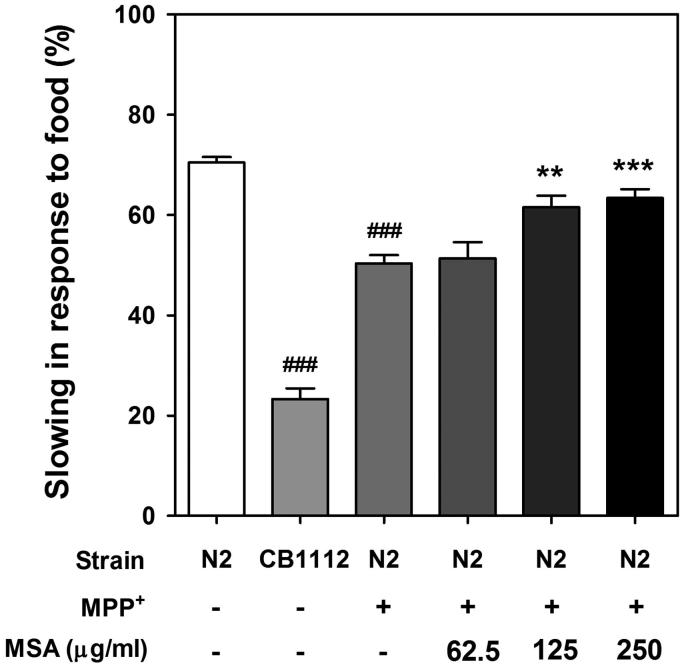
Effects of MESA on the defected food-sensing response in MPP^+^-treated *C. elegans*. Travel distances in the both of food-coated plate and non-coated plated were measured and basal slowing response were calculated. An automated behaviour-tracking system was used for tracking and recording of travel distances of worms. The CB1112 mutant strain was used as negative control. Data are expressed as mean ± S.E.M. and results are obtained from three independent assays. Significance of difference between MESA treatment and MPP^+^-treated control was determined by a one-way ANOVA. ^###^*p* < 0.001 compared with vehicle control. **p < 0.01 and ***p < 0.001 compared with MPP^+^-treated control.

In order to elucidate whether MESA-mediated DA neuroprotection was potent enough to rescue decreased DA-related functional characteristics, food-sensing assay was conducted under MPP^+^-supplemented condition. When worms come across food (OP50), they decelerate the bending frequency to feed themselves more effectively via sensing the food by DA neurons. Thus, DA neuron-degraded worm shows defected food-sensing behaviour. Herein, wild-type worms showed a decrease in travel distance ∼71.65% upon exposure to OP50, whereas worms deficient in *cat-2* (e1112) almost completely lost the slowing in response to food, as consistent with previous observation (Sawin et al. 2000). In addition, MPP^+^-treatment also appeared to reduce food-sensing response about 20%, compared to vehicle-treated worms. Interestingly, MESA-fed worms rescued DA-specific behavioural deficit in a dose-dependent manner (Figure 4).

### MESA increases the lifespan of C. elegans

**Figure 5.**
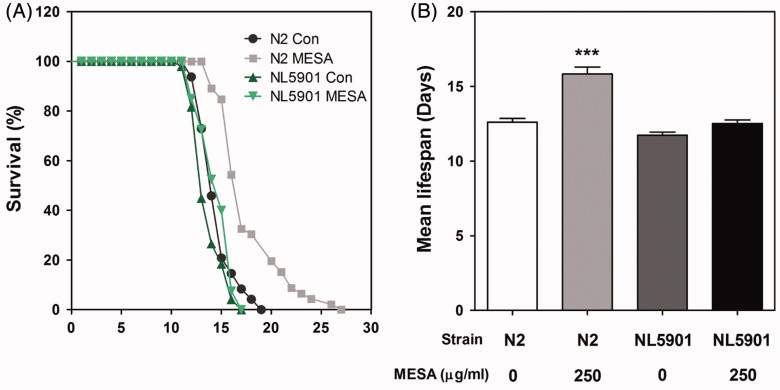
Effects of MESA on the lifespan of *C. elegans*. Worms were grown in the NGM agar plate at 25 °C in the absence or presence of MESA after embryo isolation. The mortality of each group was determined by daily counting of surviving and dead animals. (A) The lifespan of MESA-treated (250 μg/mL) N2 and NL5901 worms was plotted as a survival curve using Kaplan-Meier analysis. (B) The mean lifespan of worms was calculated from the survival curves and data are expressed as the mean ± S.E.M. Statistical difference between the curves was analyzed by the log-rank test. Differences compared to the control were considered significant at ***p < 0.001.

Since PD shares pathological features with aging, we further investigated the effect of MESA on the longevity of worms. The cumulative survival patterns was calculated by the Kaplan–Meier analysis and is shown in Figure 5(a). The mean lifespan of vehicle-treated worms was 13.55 ± 1.50 days, whereas MESA treatment efficiently prolonged the mean lifespan of wild-type worms up to 25.6% (Figure 5(b)). This result indicated that MESA possesses lifespan-extension properties as well as neuroprotective activities. However, no statistical significance was demonstrated when comparing MESA and vehicle treatment in the NL5901 transgenic worms (Figure 5(b)). This result corresponds with our observation that MESA has no effect on the α-synuclein aggregation.

## Discussion

Environmentally, damage of DA neurons can be caused by exposure to toxic substances, such as MPP^+ ^and 6-hydroxydopamine (6-OHDA). High-affinity binding of these toxins to DA transporters (DAT) explains preferential targeting of DA neurons (Schober 2004). In this study, we first tested the effect of MESA on MPP^+^-induced neurotoxicity in PC12 cells. The differentiated PC12 cells are known to have physiological properties of DA neurons (Takashima & Koike 1985). In agreement with previous studies, MPP^+  ^induced death of PC12 cells and MESA efficiently attenuated this MPP^+^-mediated cell toxicity. To further examine the protective effect of MESA in *in vivo* conditions, we investigated the survival rate against MPP^+ ^insult using *C. elegans* model system. Similar to the results in PC12 cells, MPP^+^-mediated loss of worm survival was significantly recovered by MESA supplementation.

Similar to mammals, MPP^+  ^exposure to *C. elegans* induces accumulation of MPP^+ ^in the DA neurons resulting in inactivation of mitochondrial transport chain complex I, thereby causing selective death of DA neurons (Braungart et al. 2004). Mitochondrial dysfunctions induced by MPP^+ ^ result in the generation of ROS, and finally leading to apoptosis of DA neurons via caspase activation (Liou et al. 2005; Dias et al. 2013). In this study, worms without MPP^+ ^showed vivid GFP fluorescence in all four cephalic sensilla (CEP) dendrites, while MPP^+^-treated worms displayed a significant GFP loss, indicating DA neurodegeneration. We found that this MPP^+^-mediated neuronal damage in CEP dendrites was prevented in the presence of MESA. Our unpublished data suggest that MESA possesses strong radical scavenging capacity, and thus, the neuroprotective effect of MESA could probably be associated with its antioxidant potential. However, additional study is necessary to determine the role of MESA in the caspase activation triggered by mitochondrial dysfunction.

In addition to exogenous factors favouring the formation of ROS, DA neurons are also susceptible to oxidative stress as a result of disturbance of DA metabolism (Blum et al. 2001). In *C. elegans*, *cat-2* gene encodes the nematode homolog of tyrosine hydroxylase, which is required for the DA biosynthesis. However, over-expression of CAT-2 protein in DA neurons is associated with age-dependent DA neurodegeneration (Cao et al. 2005). As previously noted, we found that DA neurons (CEPs) got damaged by excessive CAT-2 expression. In this study, CAT-2-associated DA neuronal loss was efficiently prevented in the MESA-treated worms, reflecting role for MESA in diminishing oxidative stress caused by excess internal dopamine synthesis. Thus, it seems like MESA protects DA neurons regardless of direct DAT modification, because CAT-2 overexpression, unlike MPP^+^, overproduces dopamine intracellularly. Additionally, in the current study, MESA failed to inhibit α-synuclein aggregation, suggesting MESA does not influence the chaperoning and vesicle trafficking activity. This result also supports the hypothesis that the MESA exerts neuroprotection by a DAT-independent mechanism, because α-synuclein-induced DA neurodegeneration is closely associated with DAT regulation (Cao et al. 2005; Butler et al. 2015).

Previous studies suggest that one of the roles of DA neurotransmission in *C. elegans* involves adaptive behaviour in food-sensing (Sawin et al. 2000). When worms enter the food area, CEP neurons sense surrounding bacteria and the worms and hence decrease crawling velocity to facilitate food ingestion, namely basal slowing response. In this study, MPP^+^-treated worms manifest abnormal food sensing phenotype due to damage in DA neurons. Interestingly, this abnormal behavioural phenotype was significantly recovered by MESA treatment, indicating MESA can improve not just survival but also the function of DA neuron.

Accumulated evidence demonstrated that aging may be relevant to PD (Levy 2007). Indeed, elderly PD patients showed a faster rate of motor impairment, reduced levodopa responsiveness and more severe cognitive dysfunction (Gomez Arevalo et al. 1997; Katzen et al. 1998; Yankner et al. 2008). Therefore, aging-related genes may be an effective therapeutic target for PD. Our observations of prolonged lifespan in MESA-treated worms led us to postulate the involvement of aging-related genes in MESA’s neuroprotective potential. Previously, dozens of genes that blocks α-synuclein inclusion were identified in *C. elegans* and some of them also participated in longevity regulation (van Ham et al. 2008). Herein, MESA failed to extend the lifespan of α-synuclein expressing transgenic worms, implying these genes were not required for MESA’s neuroprotective activity. However, further investigations are required to elucidate the target genes of MESA.

## Conclusions

The current study demonstrated that MESA has potent neuroprotective properties against MPP^+^-induced DA neuronal toxicity *in vitro* and *in vivo*. In *C. elegans* MESA efficiently prevented DA neurodegeneration in both MPP^+^-treated worms and *cat-2* gain of function mutants. MESA also rescued the DA specific behavioural deficit in the MPP^+^-treated worms. Additionally, MESA exhibited lifespan-extension effects in wild-type worms. In conclusion, MESA might be a valuable candidate for the treatment of PD.
